# Research on Emotion Analysis of Chinese Literati Painting Images Based on Deep Learning

**DOI:** 10.3389/fpsyg.2021.723325

**Published:** 2021-07-28

**Authors:** Jie Zhang, Yingjing Duan, Xiaoqing Gu

**Affiliations:** ^1^School of Design, Jiangnan University, Wuxi, China; ^2^Graduate School of Management, Management and Science University, Shah Alam, Malaysia; ^3^School of Computer Science and Artificial Intelligence, Changzhou University, Changzhou, China

**Keywords:** emotional analysis, Chinese literati painting, deep learning, computer vision, machine learning

## Abstract

Starting from a pure-image perspective, using machine learning in emotion analysis methods to study artwork is a new cross-cutting approach in the field of literati painting and is an effective supplement to research conducted from the perspectives of aesthetics, philosophy, and history. This study constructed a literati painting emotion dataset. Five classic deep learning models were used to test the dataset and select the most suitable model, which was then improved upon for literati painting emotion analysis based on accuracy and model characteristics. The final training accuracy rate of the improved model was 54.17%. This process visualizes the salient feature areas of the picture in machine vision, analyzes the visualization results, and summarizes the connection law between the picture content of the Chinese literati painting and the emotion expressed by the painter. This study validates the possibility of combining deep learning with Chinese cultural research, provides new ideas for the combination of new technology and traditional Chinese literati painting research, and provides a better understanding of the Chinese cultural spirit and advanced factors.

## Introduction

In ancient China, most literate people appreciated painting and calligraphy, and many well-educated people, such as Dongpo Su, were talented at painting. Because he was good at painting, poems of Dongpo Su included images depicting the fusion of poetry and painting, which can be understood as textual expression and visual performance to a high consistency. In contrast, a painter with no cultural accomplishment and whose paintings lack thought and emotion can only be called an artisan. After the Song Dynasty, almost all great Chinese painters were literati painters. The appreciation and research of literati paintings usually require researchers to have rich historical and cultural knowledge, associating the era of the author with personal behavior, emotion, knowledge, self-cultivation, feelings, and other factors (Edward, 1997; Wong, 2000; Vigneron, 2011; Bowie, 2014; Adriana, 2019). However, today, the image information of literati paintings has become the main information transmission channel for cohesive emotion. A traditional Chinese painting has complex forms and various types. In the present research, although there is no shortage of either samplings from many kinds of traditional Chinese painting forms or samplings combined with computer technology with a variety of picture characteristics for the analysis, there is still a lack of machine vision-based Chinese literati painting emotional analysis. Therefore, this study attempts to analyze the emotions of literati paintings by using artificial intelligence. We also constructed a dataset of Chinese literati paintings, visualized the characteristic area corresponding to the emotional keywords, and explored the relationship between the picture content and the expression of emotion based on machine vision to help people better understand the semantic emotion in literati painting.

### Emotional Characteristics of Chinese Literati Painting

Literati painting is the emotional product of specific groups of people in ancient Chinese society. Literati paintings are all made by literati or painters with literati nature. The special identity of the painters makes literati paintings have the nature of literati and contain the tastes of literati. However, this study does not aim at depicting the appearance of the object but pays more attention to the cultivation of extra painting work and the expression of emotions, generally through the reflection of the literatito form an indoctrination of the external society or through the expression of elegant tastes to achieve the purpose of self-entertainment. Outside the picture, the ancient literati pursued “*Lide*,” “*Ligong*,” and “*Li Yan*.” Most of the painters used painting into the Tao to obtain spiritual relief and transcendence. Painters with personal quality and sentiment strived to learn how to create a “simple” and “ethereal” mood as well as other moods to express emotions, express personality, expound philosophy, and still include the unique aesthetic taste of emotional development. Within the picture, the literati used a unique screen perspective: white background, pen and ink changes, metaphors, and other ways to express emotions with a scenery. They also used the rationality of poetry to expand the expressiveness of painting.

### Emotion Analysis of Paintings in the Context of Machine Learning

With the explosion of big data and the development of artificial intelligence, the use of advanced artificial intelligence algorithms to analyze painting works has become a major research hot spot at the intersection of art and computer vision. Current research results show that, using image analysis and feature extraction technology, computers can learn much knowledge from paintings, extract effective features of painting images, and realize painting value evaluation, painting protection, painting author identification, painting style comparison, and painting semantic emotion analysis (Prabowo and Thelwall, 2009; Xia et al., 2011; Feldman, 2013; Medhat et al., 2014; Xu et al., 2019; Ortis et al., 2020; Xue et al., 2020; Zhang et al., 2020, 2021; Castellano and Vessio, 2021; Guo et al., 2021). The analysis of traditional Chinese paintings based on computer vision technology is still in the development stage. Liu and Jiang (2014) proposed a convolutional neural network (CNN)-based feature description and a feature-weighted feature-prioritized algorithm to achieve overwhelmingly better classification performances of Chinese painting. Sun et al. (2016) analyzed the length, curvature, and density of traditional Chinese painting strokes and proposed new methods for extracting related features. Fan and Zhang (2020) reported the results of evaluating the influence of extracted features on visual order in Chinese ink paintings using a regression model.

**Figure 1:**
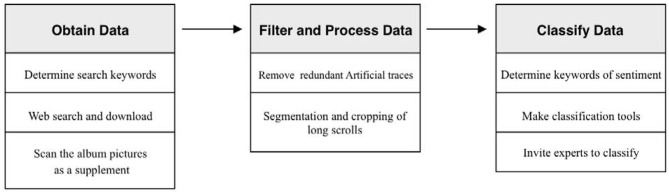
Process of making a dataset.

The process of dataset production is divided into four steps: obtaining data, filtering data, processing data, and classifying data based on sentiment analysis. Each step can be subdivided into several steps, as shown in Figure 1.

### Obtain Data

Literati paintings in the Song Dynasty were greatly developed, and a large number of famous literati painters emerged from this time period, such as Yuan Dong, Ran Ju, Cheng Li, Kuan Fan, Tong Wen, Shi Su, and Fu Mi. The literati also received much development in the Yuan, Ming, and Qing dynasties. This study selected 19 representative literati painters: Yuan Dong, Kuan Fan, Shi Su, Fu Mi, and Youren Mi from Song dynasty; Gongwang Huang, Zan Ni, Meng Wang, Zhen Wu, Mengfu Zhao, and Xuan Qian from Yuan dynasty; Shen Zhou, Zhengming Wen, Wei Xu, Chun Chen, Fu Wang, and Qichang Dong from Ming dynasty; and Tao Shi, and Bada Shanren from Qing dynasty. The image data of the representative works of the above painters were mainly obtained from network engine searching and picture album picture scanning, and the names of these painters were used as keywords for retrieving data. The picture data retrieved from the network were then downloaded and stored, and the picture album of the artist was scanned as a supplement to complete the dataset. Finally, the number of literati painting images was 635.

### Filter and Process Data

**Figure 2:**
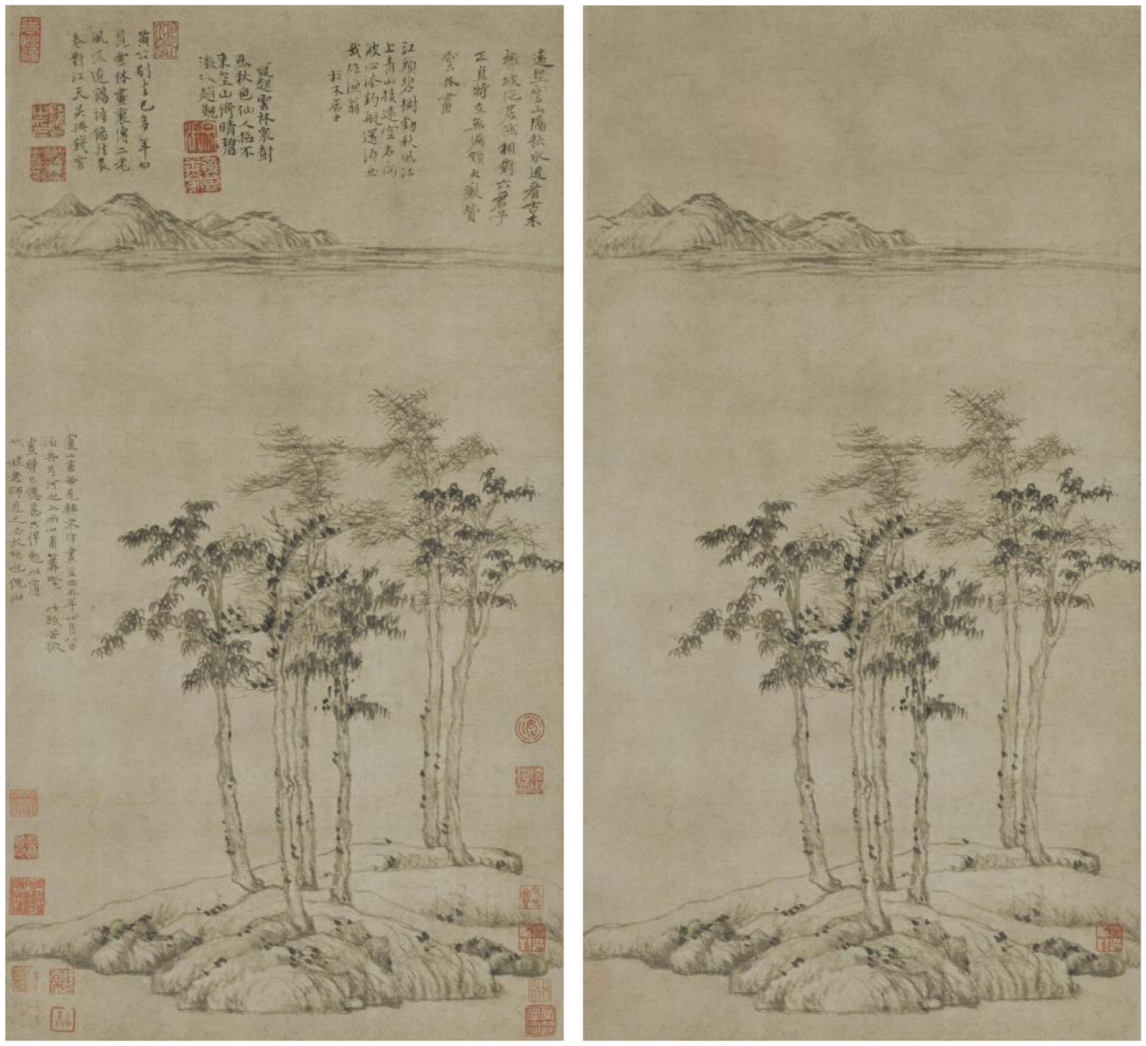
Six gentlemen figure before data processing (left) and after data processing (right).

After obtaining the literati painting data, this study next filtered and processed the data. In the acquired data, there was a large number of literati paintings with inscriptions or stamps on the white space of the paintings. The existence of these redundant artificial traces affected the original white space layout of the picture. Therefore, in the process of data processing, this study used image processing tools to remove redundant traces to restore the original appearance of the paintings, as shown in Figure 2. Additionally, to improve the accuracy of image feature extraction by the neural network, based on preserving the original blank features of the painting, this study used image processing tools to split the picture with too large aspect ratios to ensure the consistency of the pictures in the dataset on the frame. This avoided the problem of the aspect ratio of some pictures being too exaggerated affecting the overall training effect of the neural network. After filtering and processing the data, this study finally obtained 795 images to build the literati painting dataset.

### Data Classification Based on Emotion Analysis

**Figure 3:**
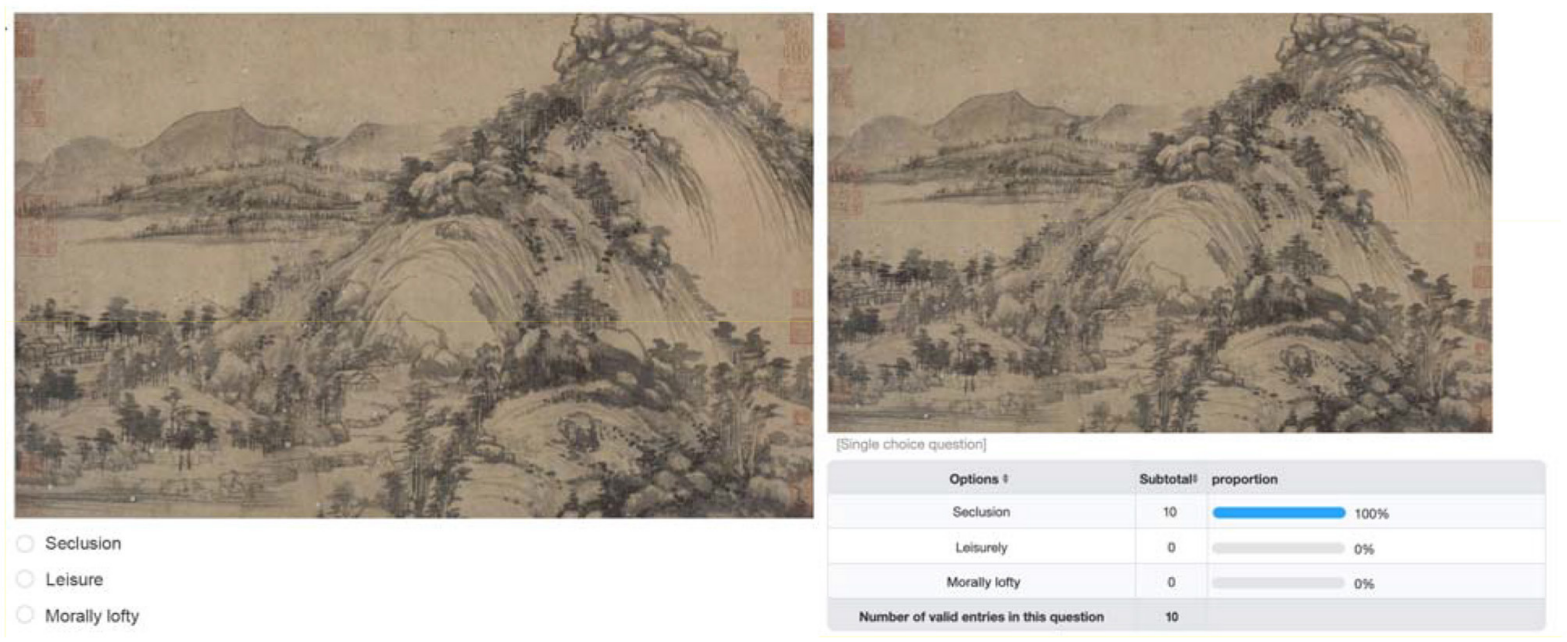
Literati painting sentiment classification system.

After filtering and processing the data, this study classified the data based on the emotional characteristics expressed in the pictures. As a typical representative of a literati painter, Shi Su emphasized that art should express his subjective emotions. He regarded painting as an important part of improving self-cultivation and lifestyle. He pursued the expressive interest of strokes instead of similarity. In terms of themes, in addition to the theme of landscape, Shi Su extended to objects such as flowers and birds, figures, plums, orchids, bamboo, and chrysanthemums—objects also endowed with noble character and emotion. On the basis of strokes, composition, and objects, for Shi Su, painting is lyrical and expresses aspirations. Therefore, the emotional characteristics that literati paintings often present are closely related to the sentiment of the painter and are usually described as open-mindedness, moral loftiness, ethereality, leisure, seclusion, and so on. This study extracts three words that are frequently used, have a wide range, and have a certain distinction between each other, namely, morally lofty, seclusion, and leisure, for classification. Then, we created a literati painting sentiment classification system in the form of a web page, so that researchers can classify and choose the option of the image while browsing the images. An example of the use of the keywords mentioned above is shown in Figure 3. After creating the emotion classification system, this study invited 10 experts in the field of literati painting to select the picture emotions in the classification system, and the option selected in the largest proportion by people for each picture is the result of emotion classification. The number of specific categories under emotional qualifiers is as follows: 282 for morally lofty, 241 for seclusion, and 272 for leisure.

## Use of CNNs to Learn and Analyze the Emotional CHaracteristics of Literati Paintings

In this study, based on the built-up literary painting emotion dataset, a CNN was used as an experimental model to identify the emotion of literati paintings. To explore the correlation between picture features and emotions under machine vision, we visualized the picture features recognized by the machine and explored the relationship between the picture features and emotions that the machine can extract through the study of the feature aggregation area.

### Improving Model Generalization Capabilities by Data Augmentation

**Figure 4:**
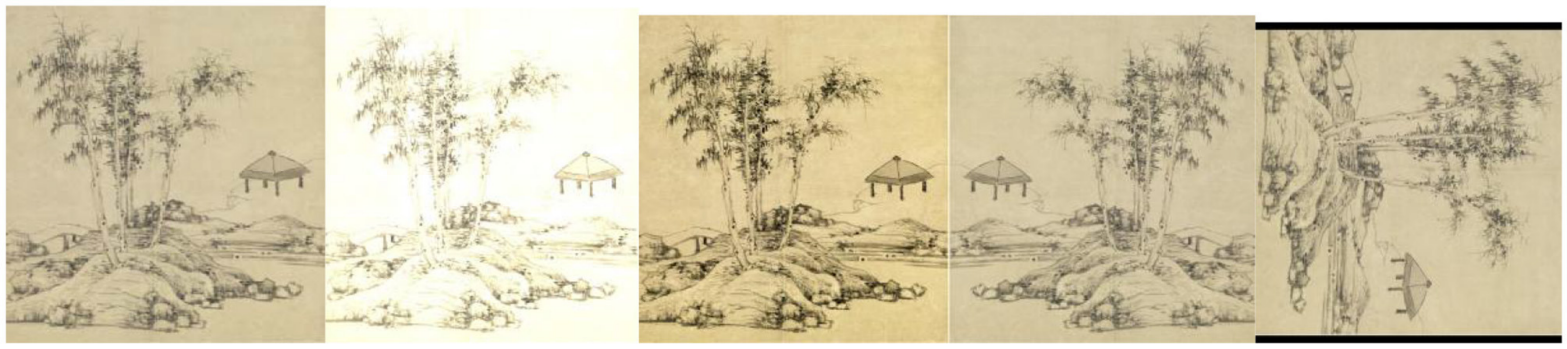
Results of data augmentation (left to right: original picture, brightness enhancement, contrast enhancement, image flip, image rotation).

Considering the limited number of training samples produced to improve the training effect, the method of data enhancement was used to improve the robustness of the model so that the collected data can be fully utilized in the model training and enhance the generalization ability of the model. In general, common data augmentation methods included brightness enhancement, contrast enhancement, image rotation, image flip, image cropping, and Gaussian noise addition. In this study, the data of each classification in the dataset were divided into a training set and a test set according to a ratio of 7:3. The data in the training set were then expanded in several common ways, namely, image brightness enhancement, contrast enhancement, image rotation, and image flip. To ensure the integrity of the picture layout and to avoid random cropping caused by missing pictures, we did not choose image cropping. In terms of brightness enhancement, an increment of 10–50% random was selected to change the dependence of the model on the brightness of a large area. In contrast to enhancement, a random increment of 10–50% was selected to change the dependence of the model on the contrast of a large area. In the aspect of image rotation, a random angle in the range of approximately −180–180° was selected for rotation so that the model can learn the object of interest from many angles and improve the robustness of the model. With regard to image reversal, each image was flipped left or right with a 50% probability to change the position of the object of interest and reduce the dependence of the model on the target position. In terms of increasing noise, increasing Gaussian noise was chosen. The effect is shown in Figure 4. As the data were expanded, the amount of data in the training set eventually increased to six times the original amount.

### Testing and Improvement of Neural Networks

#### Classic Network Analysis

After data augmentation, this study focused on the literati painting dataset using the classic networks of VGG16 (Liu et al., 2020), ResNet50 (He et al., 2016), MobileNet (Chechliński et al., 2019), GoogleNet Incepetion V1 (Szegedy et al., 2015), and GoogleNet Incepetion V3 (Szegedy et al., 2015) for sentiment classification. The test accuracies are shown in Table 1.

**Table 1:** Test accuracies of the different models.

**Model**	**Accuracy (%)**
MobileNet	38.89
GoogleNet Incepetion V1	41.82
GoogleNet Incepetion V3	45.19
VGG16	52.40
ResNet50	51.92

It can be seen from Table 1 that VGG16 and ResNet50 have better accuracy than other networks in the test of emotion classification of literature. To select a suitable model for improving the accuracy of sentiment classification step by step, this study analyzed the two models based on their characteristics. VLG16 mainly improved the detection performance by increasing the network depth. Therefore, deep network of VGG16 is more focused on a specific target than the shallow network and less focused on global information. ResNet50 introduced a residual network, which deepened the network depth and was able to integrate the shallow information into the deep information so that the model could utilize the global information of the picture to some extent. In summary, due to the overall effect of Chinese literati painting on the expression of emotion, this study chose ResNet50 to obtain better test results in the classification and recognition of the emotion of literati painting.

#### Improvement of the ResNet50 Network

For deep learning networks, the model effects are theoretically better and more expressive as the number of layers in the network increases. However, it has been found that, when the CNN network reaches a certain depth and continues to deepen the network, the classification performance will not improve. Rather, there is slower network convergence and lower accuracy. Thus, even if the problem of overfitting is solved by extending the dataset, the classification performance and accuracy cannot be improved. In 2015, when Kaiming et al. found that a residual network could solve the problem, they proposed ResNet50.

**Figure 5:**
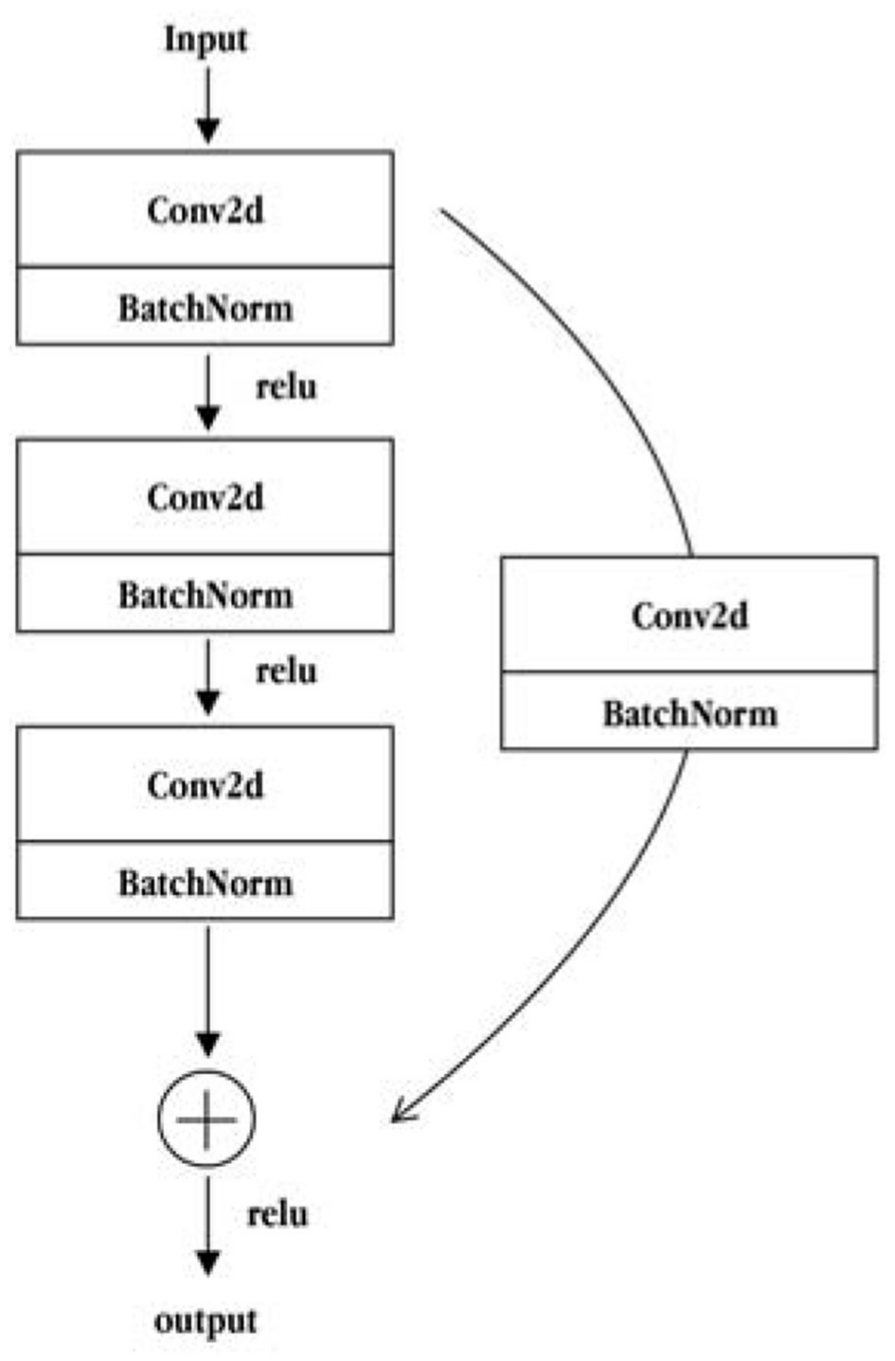
The Conv Block module structure diagram.

**Figure 6:**
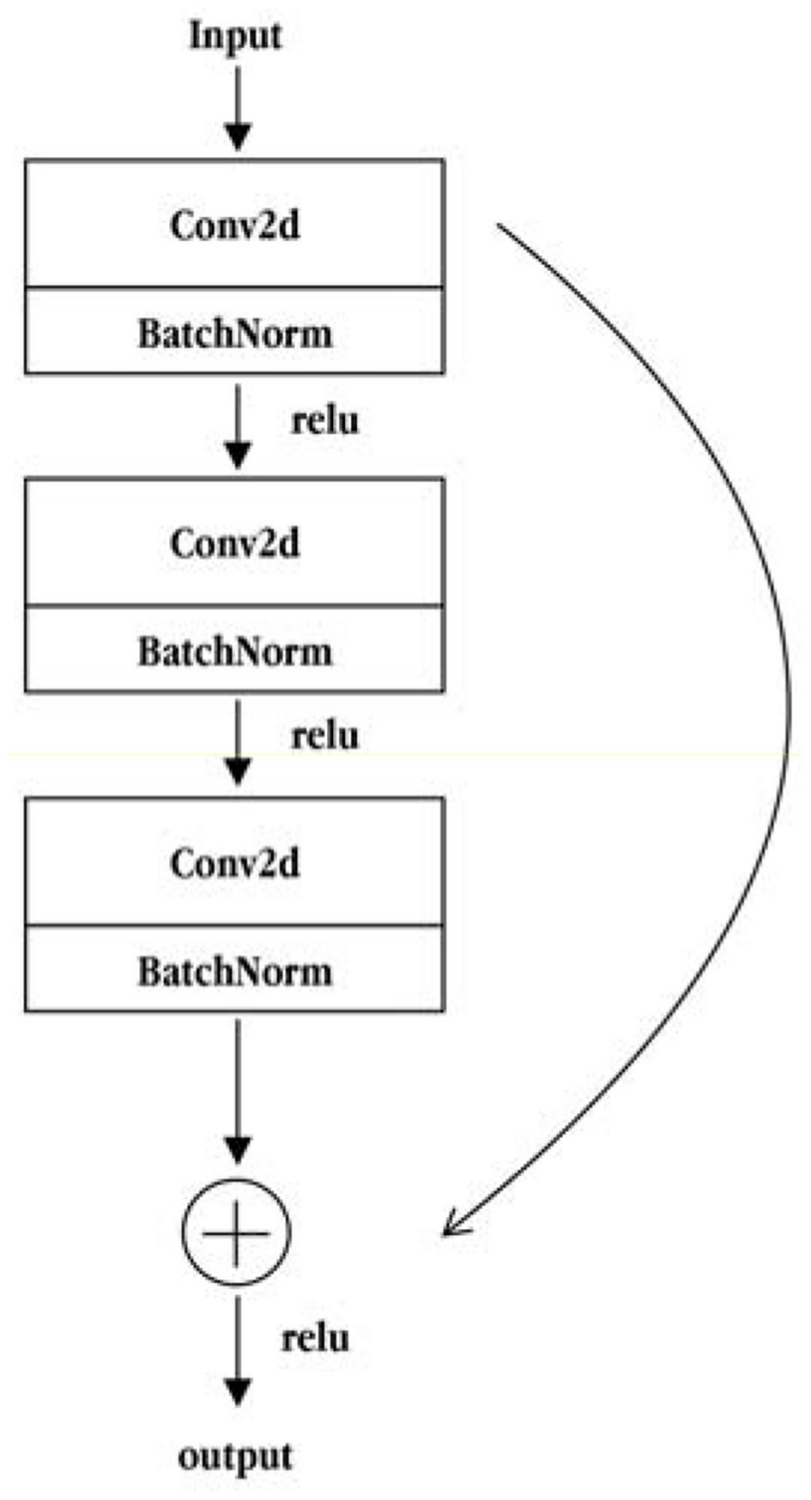
Structural diagram of the Identity Block module.

The ResNet50 network is composed of two basic residual modules: the Conv Block and the Identity Block. The structure of the Conv Block is shown in Figure 5. From the figure, it can be seen that the Conv Block consists of two parts. The left part is the main part, which is composed of the two convolution operations, standardization and ReLU activation function blocks, and one convolve and standardization block. The right part is the residual edge part, which is composed of a convolution block and a standard block. Then, the main part is added to the residual side part and the output is obtained by non-linear processing of the ReLU activation function. The residuals of the Conv Block go through a convolution operation that causes the input and output dimensions to be inconsistent, so they cannot be concatenated continuously. Their purpose is to change the dimensions of the network. The structure of the Identity Block is shown in Figure 6. As seen from the figure, the Identity Block is composed of two parts: the left part is the main part, which is consistent with the Conv Block. The right part is the residual side part, which adds directly to the trunk output without any processing and then outputs it through the non-linear processing of the ReLU activation function. The entity block input dimension is the same as the output dimension and can be concatenated to deepen the network. Finally, based on the above two types of residual modules, the Conv Block and the Identity Block constitute a complete ResNet50 network. The core modules are composed of 3, 4, 6, and 3 residual modules using the Conv Block module 4 times and Identity Block 12 times.

**Figure 7:**
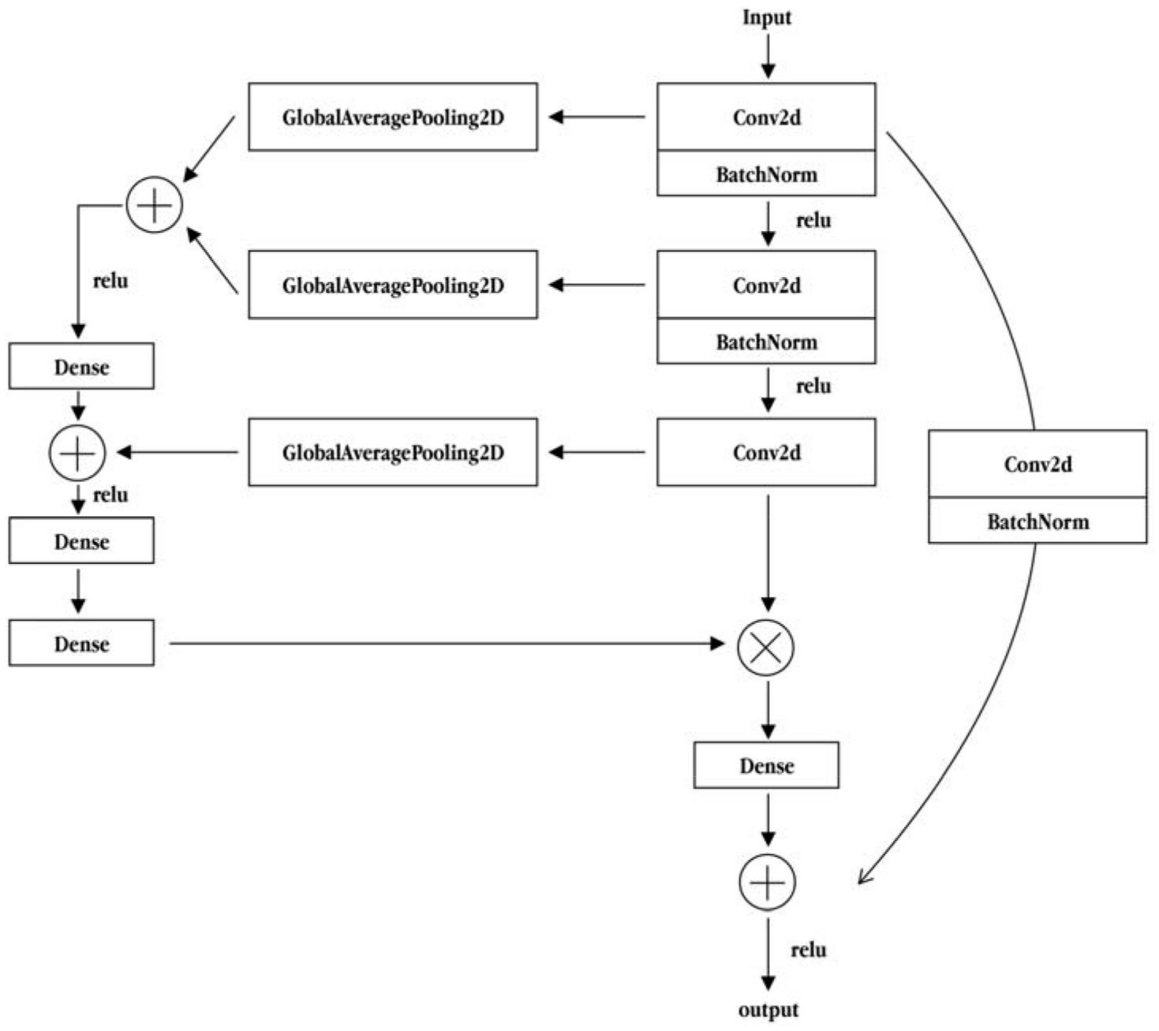
Convolutional layer feature fusion residual module of backbone part.

To improve the accuracy of the emotional classification of literary painting, this study referred to the method of feature recalibration in deep network aggregation (Wang et al., 2021) to improve the main part of the residual module. This was done by converging the main part of the multiple convolution layer characteristic information, and then the information of the integration of each layer is fed back to the output of the main part for reintegration. The process is shown in Figure 7. After global average pooling, the residual module and the main part of the first two convolution layer characteristic information merges. The merged vector dimension of the fully connected layer are combined with the globally averaged feature information of the third convolution layer. Then, they form a bottleneck structure through a lower-dimensional full-connection layer and a layer-up full-connection layer to enhance the feature information extraction ability. Finally, the characteristic information of the last three convolutional layers is used as the weight, and the third convolution layer output information of the main part is integrated to form the final main part convolution layer characteristic fusion residual module. As shown in Figure 7, on the basis of the residual module, the main convolution layer feature fusion residual module integrates the three-layer convolution layer information of the main dry part of the module to feeds it back to the output of the main edge. This further enhances the use of deep information to integrate shallow information for better analysis of the emotion of the literati painting.

Based on the main coil feature fusion residual module proposed above, three sets of improvement schemes were adopted: feature fusion improvement only for the Conv Block module, feature fusion improvement only for the Identity Block module, and feature fusion improvement for both modules at the same time. The resulting improvement is shown in Table 2.

**Table 2:** Three improvement schemes and their model accuracies.

**Improve Proposals**	**Accuracy of Model (%)**
Feature fusion is only performed on the Conv Block module	54.17
Feature fusion is only performed on the Identity Block module	54.17
Feature fusion of two modules at the same time	50.93

It can be seen from the data in Table 2 that only the feature fusion of the Conv Block module and only the feature fusion of the Identity Block module exhibited a certain improvement in the optimal model accuracy, which is 2.25% more accurate compared to the classic ResNet50, and 1.77% more accurate compared to the classic VGG16. However, when the two modules are optimized for feature fusion at the same time, the optimal model accuracy is lower than the accuracy of the classic model. This phenomenon may be due to the repeated reuse of shallow information in the network so that deep semantic information is more focused on specific objectives. Thus, better accuracy cannot be achieved.

**Figure 8:**
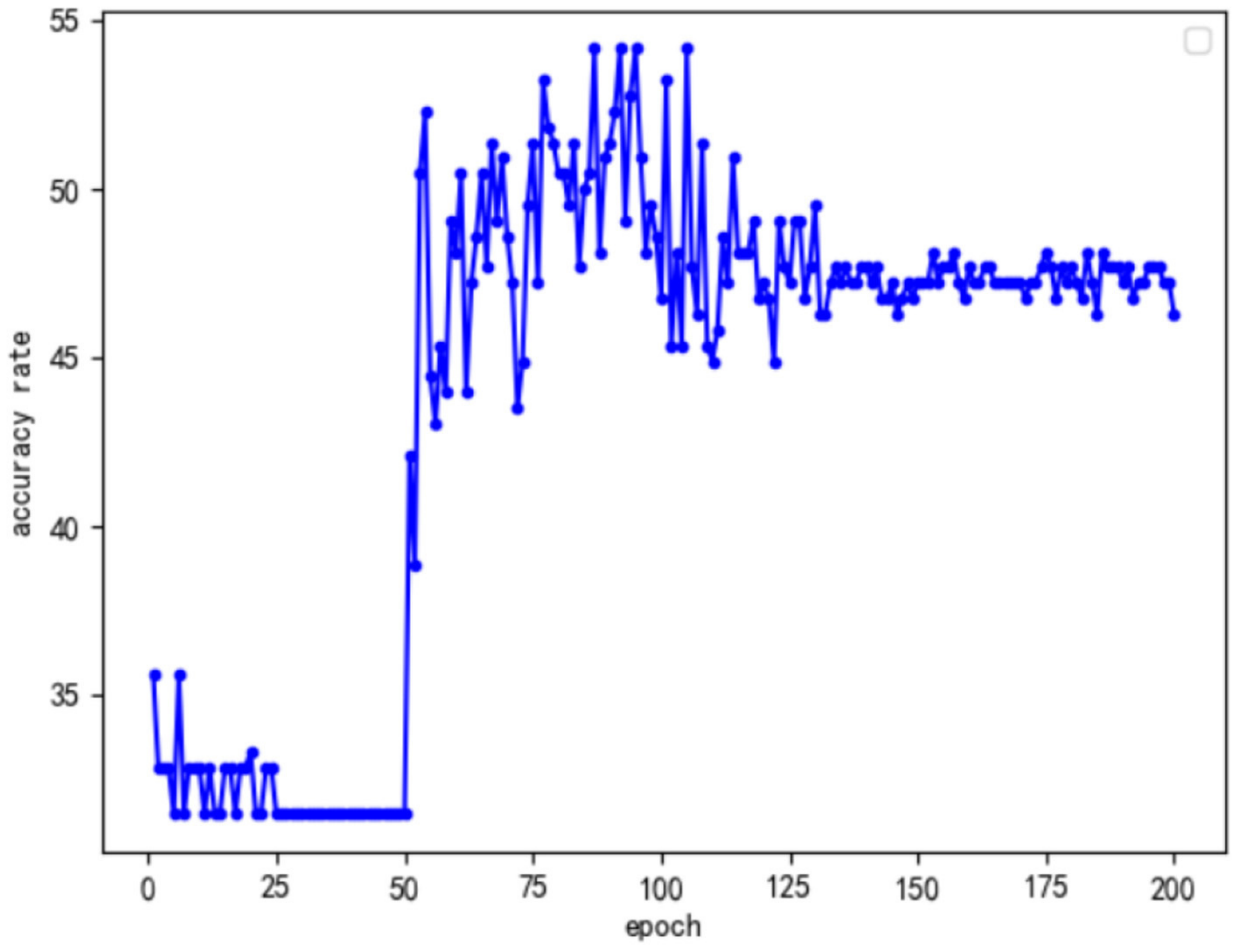
Only perform feature fusion model accuracy curve for the Conv Block module.

**Figure 9:**
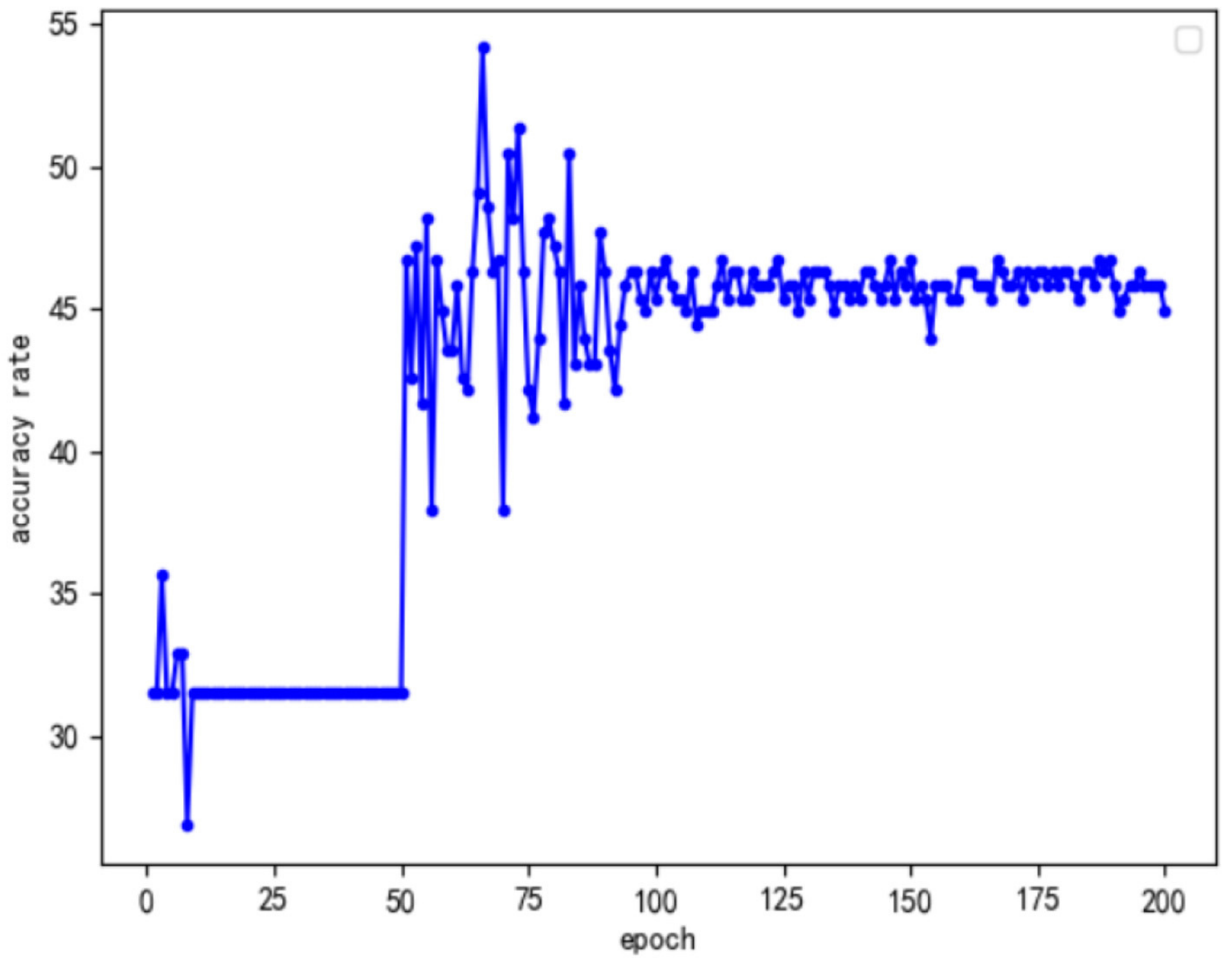
Only perform feature fusion model accuracy curve for the Identity Block module.

In addition, to explore why the optimal accuracy rates of the first two improved models were the same, this study analyzed the change curves of the accuracy of the two types of improved models to evaluate the stability of the two improved models. The curves of the accuracy of the two types of improved models with the number of training rounds are shown in Figures 8, 9.

By analyzing the above two kinds of curves, we found that they have some common characteristics. First, the accuracy of the first 50 rounds of training was low because, in order to speed up the efficiency of training in the first 50 rounds that used the frozen training ideas, only part of the data training played a warmup and preparation role. Another common feature was that, after 100 rounds of training, the accuracy of the model tended to stabilize; however, this was not the optimal model, and analysis may suggest increasing the number of model training rounds. On the one hand, the model exhibited the overfitting phenomenon; on the other hand, the model of a specific target understanding further deepened so that the overall information of the model, with the increase in the number of training rounds, was decreased. Based on the above analysis, this study focused on the training between round 50 and round 100, and the results are as follows: Comparing the model accuracy of the two types of optimization models during the 50 to 100 rounds, it was found that only the Conv Block module feature fusion model accuracy was stable, as there were many peaks of optimal accuracy. Furthermore, only for the accuracy of the identity block module feature fusion model, after the first peak with an increase in the number of epochs, the overall model accuracy showed a clear downward trend. Combined with the analysis of the structure of ResNet50, it was not difficult to see that there were 16 residual modules in the ResNet50 network, only 4 Conv Block modules and 12 Identity Block modules. Thus, as the number of trainings increased, the Identity Block module was more affected by specific targets than the Conv Block module. Given the effect of the Conv Block module in increasing the network dimensions, the Identity Block module has the effect of increasing the depth of the network. In view of the above analysis, it can be concluded that, in future research, we can improve the neural network model from the perspective of improving the model dimensions in order to achieve a better emotional analysis of the effect of literati painting.

### Visualize the Feature Gathering Area

This study proposes a new method of art image derivative design based on emotion analysis. First, the significance of personalization was analyzed in the design of art derivatives. Then, the problems existing in the personalized design method were pointed out, the new needs of users in the background of intelligent technology were analyzed, new design ideas combined with new technology were put forward, the main technical principles of the technology were analyzed, and practical solutions were proposed to explore the new design paradigm. Finally, by constructing a personalized design system of image derivatives driven by user facial emotion data based on the paintings of Van Gogh, this study verified the feasibility of the solution. It was found that the innovation of the design paradigm is imperative. It was significant for us to change the original design paradigm and introduce new technology, which could improve the design using current new technology and better serve the needs of the public.

To explore the cognitive basis of the emotional analysis of Chinese literati paintings by machines from computer vision, we visualized the characteristic areas related to emotional keywords in the sample of the paintings of literati according to the model visualization method, Guided Grad_CAM (Selvaraju et al., 2020), and then explored the relationship between the content and emotion of the picture from the computer vision. The Grad_CAM process of visualizing the emotional characteristics in the sample of literati painting consisted of the following steps. First, the image was obtained after the last convolution of the feature extraction. In this study, the feature map was the final layer of the ResNet50 convolution layer res5c_branch2c. Then, we selected the node with the highest softmax value, the category with the highest confidence to reverse propagation, found the gradient for the last convolution layer, and took the mean gradient of each feature map as the weight of the feature map. Next, we multiplied each feature map by the weight to obtain the weighted feature map, activated it with the ReLU activation function, normalized it, and obtained the thermal effort (Grad_CAM heatmap). The ReLU function was chosen to remove feature diagrams that contributed negatively to classification, i.e., useless features, and to preserve only feature maps that have a positive contribution to feature classification, so that the resulting positioning map only reflected the category features with the highest confidence. The softmax value was then guided through backpropagation, that is, when propagation was reversed, only values with inputs and gradients >0 were passed, which resulted in a feature image (G-B image) containing deep semantic information. Finally, the thermal effort (Grad_CAM heatmap) and the feature image (G-B image) containing deep semantic information were merged to form the final Guided Grad_CAM image.

**Figure 10:**
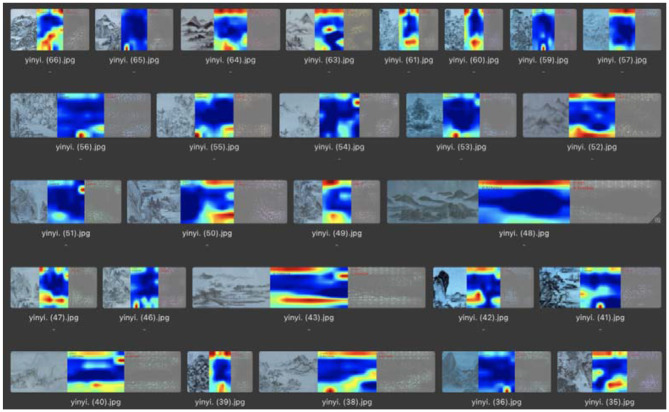
Feature visualization results in the “seclusion” category.

**Figure 11:**
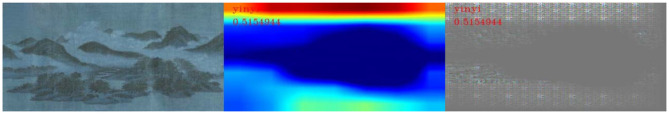
Sky and river corresponding to the “seclusion” feature.

After completing the training of the model, the pictures in the test set were put into the model for emotional classification, the characteristic gathering area of the correct images was visualized, and the connection between the picture content and the expression of emotion was explored by analyzing the visualization results. The visualization results were analyzed as follows:

In the recognition of “seclusion,” the number of test pictures was 68, and the number of correct classifications was 55. In this category, the visualized significant feature areas were mainly concentrated at the top and bottom of the picture, each occupying approximately a quarter of the entire area, which also corresponded to the whitening in the picture. Thus, it can be concluded that the recognition of the “seclusion” emotion in the picture by the machine is mainly based on the analysis of the location and area of the white areas left in the picture to obtain the classification results, as shown in Figure 10. A comprehensive analysis concluded that, in the machine vision of the literary painting emotion analysis, the expression of the “seclusion” emotion of the painting at the top and bottom of the canvas each have a quarter of its size in white. Combined with the semantic analysis of the literati painting, these areas are mostly sky and river, as shown in Figure 11.

**Figure 12:**
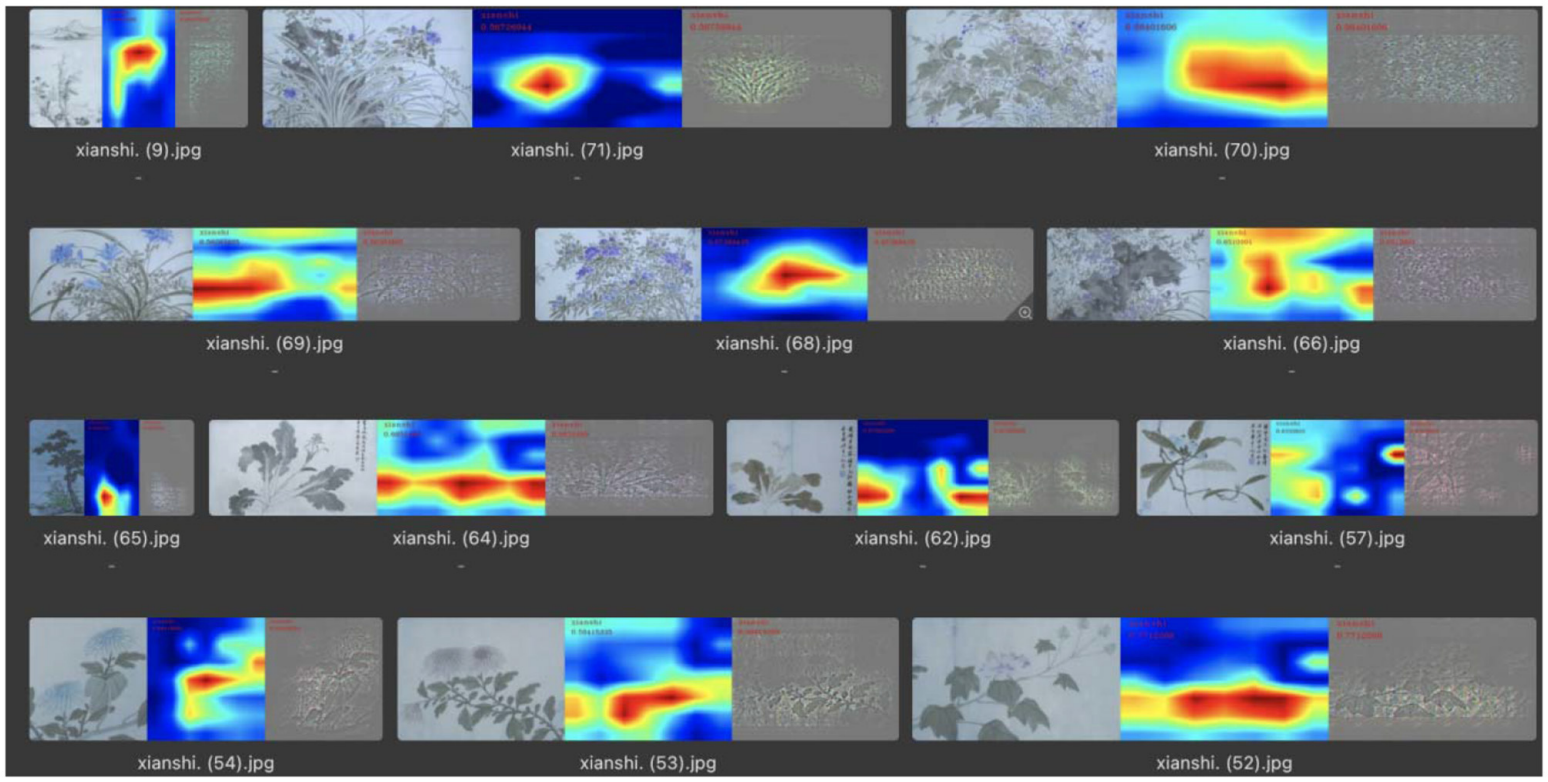
Feature visualization results in the “leisure” category.

**Figure 13:**
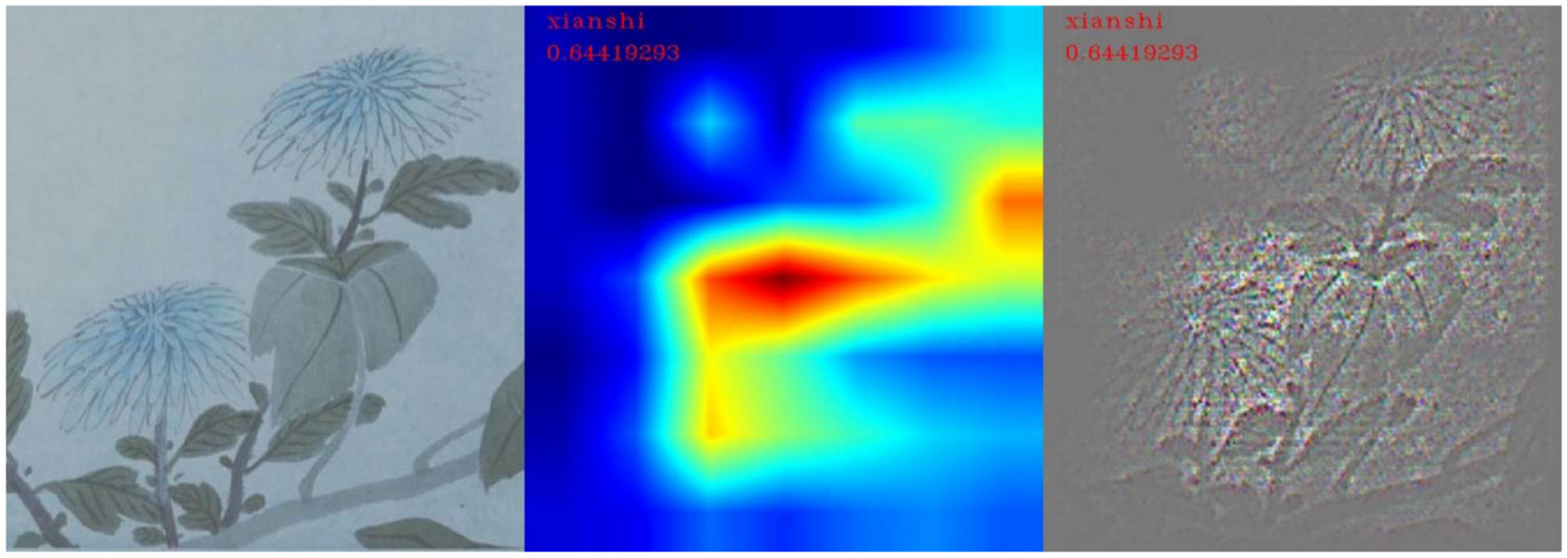
Flowers and leaves corresponding to the “leisure” feature.

In the identification of “leisure” emotions, the number of test pictures was 63, and the number of correct classifications was 32. In this category, the visualized area of remarkable features was mainly concentrated on the objects in the picture, so the extraction of the features of the picture by neural networks was mainly in the recognition of objects in the picture, as shown in Figure 12. In the correctly classified test images, the main objects in the picture labeled “leisure” were mostly flowers, birds, rocks, and plants with leaves, as shown in Figure 13. Therefore, the machine mainly recognizes the abovementioned objects to determine whether it is a “leisure” emotion.

**Figure 14:**
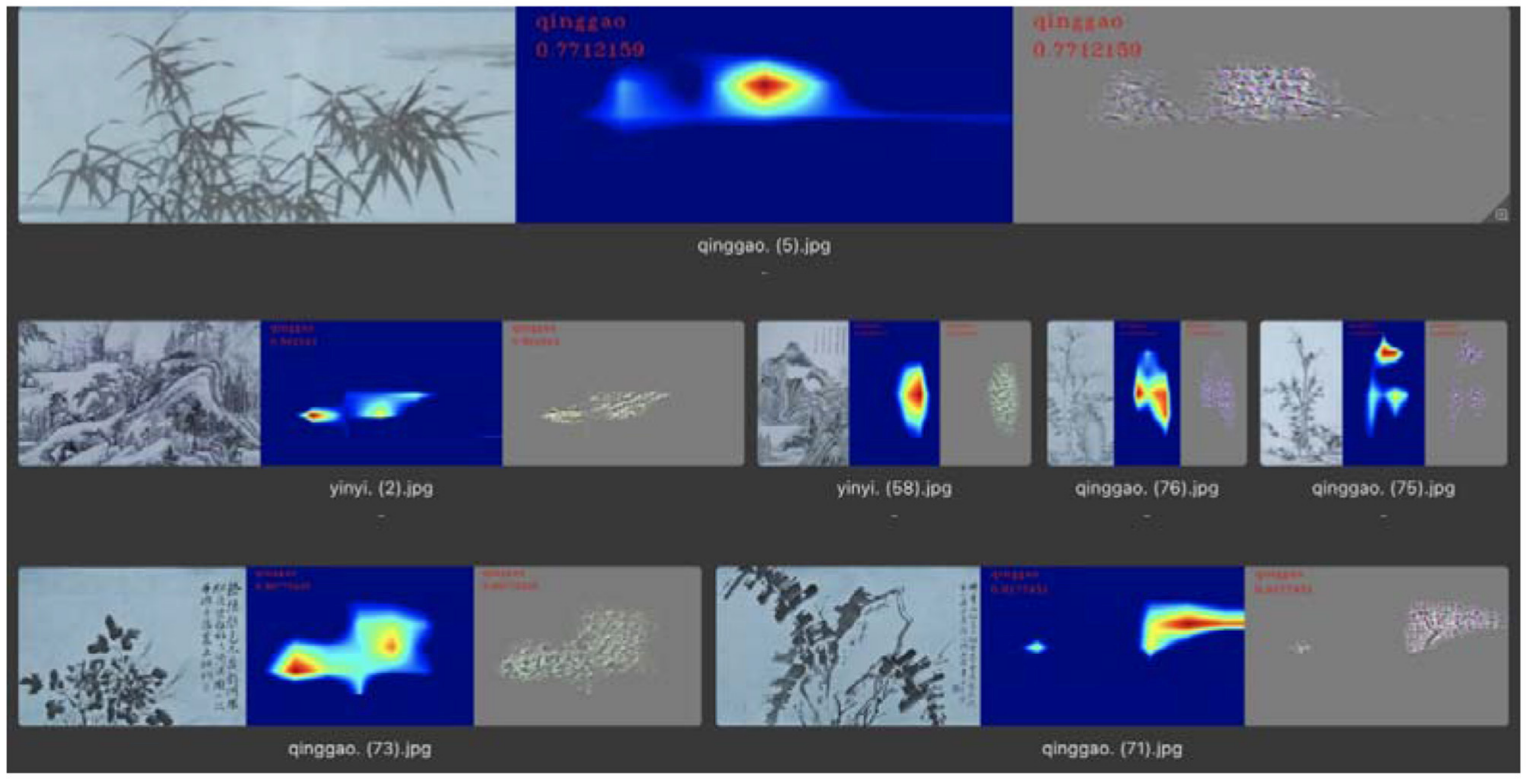
Feature visualization results in the “morally lofty” category.

In the identification of the “morally lofty” emotion, the number of test pictures was 71, and the number of correct classifications was 19. Because of the similarity between the picture features of “seclusion” and “morally lofty,” the effect of the machine in the emotional classification was not good, and the paintings expressing “morally lofty” emotion were often misjudged as “seclusion,” thus affecting the visualization of features under the category of “morally lofty.” However, an analysis of a small number of classified and accurate graphs revealed that the visualized area of distinctive features was the center of the main concentrated picture and that the area corresponded to the blank in the picture, as shown in Figure 14.

In summary, through the results of visual analysis, it can be concluded that, in paintings expressing “seclusion” emotions, the feature extraction of the machine is mainly concentrated in the blank part of the screen, and the top and bottom of this type of screen both occupy ~4% of the screen area. In one part of the blank spaces in the paintings that express “leisure” emotions, the feature extraction of the machine was mainly concentrated on the main objects in the picture. The main objects in this type of picture were generally flowers, birds, rocks, and plants with leaves. In the paintings expressing the emotion of “morally lofty,” the extraction of features by the machine was mainly concentrated in the blank part of the picture. In this type of painting, there was a small blank area in the center of the picture.

## Conclusion

This study combined existing machine learning technology with a self-built Chinese literati painting sentiment dataset to train five classic neural network models, VGG16, ResNet50, MobileNet, GoogleNet Incepetion V1, and GoogleNet Incepetion V3. Through a comparison of the accuracy of models, ResNet50 was selected as the most appropriate model for the emotion classification of literati paintings. Then, based on the multilayered aggregation feature of the recalibration algorithm, the study improved ResNet50 and obtained three models with different improvement schemes. By visualizing the accuracy under different improved methods after each round of training, it was found that the improved Conv Block model was the most stable. Finally, the present study put the images in the test set into the trained model for emotion classification and visualized the salient feature regions of the correctly classified pictures. By summarizing the rules, a series of picture contents and the connection relationship between the sentiments the paintings expressed were obtained.

Although the existing research results have been effective, there are still areas for improvement. First, due to the limited number of pictures in the literati painting sentiment classification dataset, the training effect of the model was greatly reduced, which resulted in the accuracy of the model being unable to meet expectations. In addition, finding a more appropriate data augmentation method can improve the accuracy of the emotion classification of the model. Therefore, in subsequent research, researchers can improve the performance of the dataset by improving the richness and professionalism of the dataset, comparing multiple parameters, and choosing the best data augmentation method to improve the performance of the dataset, thereby training the generalization ability to be a stronger literati painting sentiment of the classification model. At the same time, by improving the model of the neural network from the perspective of increasing the model dimension, the accuracy of the model can be improved.

Second, the effect of the existing feature region visualization was still not sufficiently ideal to explore the differences in the content of pictures with similar characteristics in different classifications and find the reasons for the above problem. Therefore, in future research, researchers can enhance the ability of the model to visualize the extracted features to obtain a clearer calibration map of salient feature regions. With this, we can better summarize and analyze literati painting images under different expressions of sentiments, which helps us to more accurately understand the connection between the content of the image and the sentimental expression from machine vision. In addition, this type of method can be used for the emotional analysis of paintings of other styles, for the exploration of the inner relationship between the aesthetic laws and emotional expression of the creator through the assistance of computer vision and for assisting people in understanding the emotion of paintings in a variety of styles. Furthermore, in addition to being connected with emotions, the characteristics of paintings can be connected with external factors such as the life experience of the creator and social events affecting artistic creation. This can help researchers to explore the relationship between the characteristics of the picture and the reasons for the mutual influence between the characteristics of the picture and the external factors. The sentiments of literati paintings are confusing, but this special group of literati mastered the highest culture and the highest thoughts in ancient China. Therefore, literati paintings, as sentimental products of the literati, must convey the culture, thoughts, and emotions of ancient Chinese society. The study of literati painting helps to understand the spirit of Chinese culture and art at a deeper level and helps to grasp the artistic characteristics and advanced factors of Chinese painting as a whole.

## Data Availability Statement

The datasets presented in this study can be found in online repositories. The names of the repository/repositories and accession number(s) can be found below: https://github.com/zzj-dyj/classification-of-literati-painting-sentiment/tree/master/datasets.

## Author Contributions

JZ, YD, and XG developed the theoretical framework and model in this study and drafted the manuscript. YD implemented the algorithm and performed the experiments and result analysis. All authors contributed to the article and approved the submitted version.

## Conflict of Interest

The authors declare that the research was conducted in the absence of any commercial or financial relationships that could be construed as a potential conflict of interest.

## Publisher's Note

All claims expressed in this article are solely those of the authors and do not necessarily represent those of their affiliated organizations, or those of the publisher, the editors and the reviewers. Any product that may be evaluated in this article, or claim that may be made by its manufacturer, is not guaranteed or endorsed by the publisher.
